# The Development of Psychiatric Services Providing an Alternative to Full-Time Hospitalization Is Associated with Shorter Length of Stay in French Public Psychiatry

**DOI:** 10.3390/ijerph14030325

**Published:** 2017-03-21

**Authors:** Coralie Gandré, Jeanne Gervaix, Julien Thillard, Jean-Marc Macé, Jean-Luc Roelandt, Karine Chevreul

**Affiliations:** 1 ECEVE, UMRS 1123, Université Paris Diderot, Sorbonne Paris Cité, INSERM, 75010 Paris, France; jeanne.gervaix@urc-eco.fr (J.G.); julien.thillard@urc-eco.fr (J.T.); jroelandt@epsm-lille-metropole.fr (J.-L.R.); karine.chevreul@urc-eco.fr (K.C.); 2 AP-HP, URC Eco, DHU PePSY, 75004 Paris, France; 3 National Conservatory of Arts and Crafts, LIRSA, EA 4603, 75003 Paris, France; macejmarc@gmail.com; 4 World Health Organization Collaborating Centre for Research and Training in Mental Health, 59000 Lille, France

**Keywords:** length of stay, alternatives to full-time hospitalization, psychiatry, mental health services, environmental characteristics

## Abstract

International recommendations for mental health care have advocated for a reduction in the length of stay (LOS) in full-time hospitalization and the development of alternatives to full-time hospitalizations (AFTH) could facilitate alignment with those recommendations. Our objective was therefore to assess whether the development of AFTH in French psychiatric sectors was associated with a reduction in the LOS in full-time hospitalization. Using data from the French national discharge database of psychiatric care, we computed the LOS of patients admitted for full-time hospitalization. The level of development of AFTH was estimated by the share of human resources allocated to those alternatives in the hospital enrolling the staff of each sector. Multi-level modelling was carried out to adjust the analysis on other factors potentially associated with the LOS (patients’, psychiatric sectors’ and environmental characteristics). We observed considerable variations in the LOS between sectors. Although the majority of these variations resulted from patients’ characteristics, a significant negative association was found between the LOS and the development of AFTH, after adjusting for other factors. Our results provide first evidence of the impact of the development of AFTH on mental health care and will provide a lever for policy makers to further develop these alternatives.

## 1. Introduction

The burden of mental disorders worldwide is high. They will affect one in three individuals over the course of their lifetime [1], and are anticipated to become the leading cause of disability-adjusted life years by 2020 [2,3]. In France, national prevalence data are scarce, but it is estimated that mental disorders contribute to 14% of the overall disease burden, with mental illness constituting the leading cause of disability [4,5], and the suicide rate is among the highest in Europe [6]. Moreover, the costs associated with mental disorders are considerable. They account for 8% of the total national health spending and represent the first item of expenditures for Statutory Health Insurance [7,8].

A major challenge of the mental health care system in France is providing optimal care to confront this epidemiological and economic burden. This system is characterized by a territorial organization into geo-demographic areas (sectors) where multidisciplinary teams enrolled and paid by a hospital coordinate and supply inpatient and outpatient services, including ambulatory care and community-based care, to cover the mental health needs of their population. Sectors are the cornerstone of the organization of public mental health care delivery which represents nearly 70% of the costs of psychiatric care in France [8]. Historically, they have relied mostly on inpatient care for the treatment of mental disorders, but this model has been questioned, in particular because of its costs and patients’ dissatisfaction [9,10]. Following recent international recommendations [11,12,13], several countries have extensively developed alternatives to full-time hospitalizations for inpatients (AFTH) [14,15,16,17,18]. AFTH encompass ambulatory care, part-time hospitalizations (day or night care, part-time therapy centers and therapeutic workshops) as well as full-time care outside of inpatient settings integrated in the community, i.e., hospitalizations at home, stays in therapeutic apartments, stays in specially trained families, crisis centers and rehabilitation centers. As a result, several different kinds of staff can work in those alternatives. They include psychiatrists and other medical doctors, nurses, nursing auxiliaries, psychologists, physiotherapists, social and educational staff as well as administrative staff. In France, the development of AFTH is still limited [19], despite support from policy makers [20]. An assessment by the French National Court of Auditors has shown that the implementation of AFTH was slowed down by the resistance of health professionals [21]. This is possibly due to a lack of consensus regarding the benefits of AFTH among the different schools of thought in the mental health field [21].

In parallel, international recommendations for mental health care have advocated for a reduction in the length of stay (LOS) in full-time hospitalization [22,23] as prolonged hospitalizations can result in isolation and loss of autonomy, and are unpopular among patients [13,24,25,26]. The development of AFTH could facilitate alignment with those international recommendations through two main mechanisms. First, previous international research has shown the benefit of AFTH, in particular in terms of increased quality of life, clinical outcomes, adherence to treatment, accessibility and continuity of care [27,28,29,30,31,32,33]. This suggests that AFTH have the potential to decrease patients’ severity of illness through increased quality of care. As a consequence, when their development is satisfactory, patients will only require full-time hospitalizations for a limited period of time. Second, it has been widely demonstrated that health care supply influences practice [34,35]. The lack of AFTH could therefore result in an increased length of stay (LOS) in full-time hospitalization when no satisfactory option is available at the end of a patient’s full-time hospitalization. Physicians in hospitals with more AFTH may be more inclined to discharge inpatients earlier because they know AFTH are available and can provide alternatives to patients in full-time hospitalization well enough to be discharged but not well enough to be sent home without further care [36].

There is currently a dearth of research to assess if the development of AFTH does result in reduced LOS and previous work has advocated for more research in that field worldwide [37]. However, when studying the impact of AFTH on LOS, a wide range of factors, which may also be associated with LOS, should be considered. They include patient, health care provider and environment characteristics. Some of them can influence LOS through similar mechanisms as AFTH provided within psychiatric sectors. Indeed, some factors can impact the patient’s health status and readiness for discharge, in particular clinical factors such as diagnosis, symptoms severity and comorbidities [38,39,40,41] while some factors can be associated with early discharge such as the implementation of discharge planning or the availability of medical and social care in the community [38,41,42,43]. In addition, associations were shown between the LOS and patients’ demographics and socio-economic characteristics [38,40,41] as well as either institutional characteristics (such as specialization or teaching status) or organizational characteristics (such as number of beds) of the health care provider [38,44].

In this context, the objective of our study was to evaluate whether the development of AFTH in French psychiatric sectors was associated with a reduction in the LOS in full-time hospitalization, taking into account the other factors potentially associated with the LOS.

## 2. Materials and Methods

A retrospective study was carried out using the French national discharge database (*Recueil d’informations médicalisé en psychiatrie*, RIM-P) [45], which records all hospital stays and outpatient care contacts in psychiatric hospitals, the annual national survey on health care providers (*Statistique annuelle des établissements*, SAE) where hospitals report their activity in a declarative manner for the past year [46], and other databases non-specific to psychiatry.

### 2.1. Setting

Psychiatric care in public and private non-profit hospitals performing public service duties in France is delivered by sectors. They are multidisciplinary teams enrolled and paid by a hospital in charge of providing care to the population of a given geo-demographic area, either through ambulatory care, part-time hospitalization, full-time hospitalization or full-time care outside of inpatient settings. The catchment areas of psychiatric sectors are relatively homogenous in size across France, except in overseas French territories where they are larger. Sectors therefore represent an optimal unit of analysis of variations in psychiatric care as they remain the cornerstone of the organization of public mental health care delivery in France both for inpatient and outpatient care. There are specific sectors—with differing organizations—for adult, child and adolescent, and forensic psychiatry. Given these elements and to ensure comparability, our study focused on psychiatric sectors at public and private non-profit hospitals that perform public services in mainland France and provide care for adult patients outside of forensic settings.

### 2.2. Study Population

We included patients in full-time hospitalization whose care was reported in the RIM-P for the year 2012 (most recent year available at the start of the study) and who were diagnosed with a mental disorder from Chapter V of the International Classification of Diseases, tenth revision (ICD-10) [47], excluding organic mental disorders (F00-F09), mental retardation (F70-F79) and psychological development disorders (apart from pervasive developmental disorders) (F80, F81-F83, F88-F89). This diagnosis scope corresponds to the scope of psychiatrists’ expertise in France and has been used in previous studies on mental health [48,49]. Patients with at least one diagnosis of mental disorder outside of this scope were excluded from the analysis.

As the databases used for the study were not totally exhaustive for the year 2012 and in order to ensure data quality, we further excluded patients seen in sectors belonging to a hospital which: (i) did not report consistently its annual full-time inpatient activity and/or its number of psychiatric sectors in the RIM-P and the SAE databases; (ii) did not report its outpatient activity; or (iii) did not report data requested to assess the development of its AFTH as described below.

### 2.3. Development of AFTH

There is no direct measure of the development of AFTH. One way to estimate it is to determine the share of human resources allocated to those alternatives out of the total human resources allocated to psychiatry in a given hospital. Human resources indeed represent 70% of the hospital budget for the treatment of somatic illnesses [50,51] and it is estimated that this percentage is even higher for the care of psychiatric disorders [52,53,54]. The development of AFTH was therefore estimated by the ratio of the number of full-time equivalents (FTEs) of staff working in departments providing alternatives to full-time hospitalizations over the total number of FTEs in the hospital to which each sector belonged, i.e., (total number of FTEs—total number of FTEs in full-time hospitalization)/total number of FTEs. All kind of AFTH provided by psychiatric sectors (ambulatory care, part-time hospitalizations and full-time care outside of inpatient settings) and all kind of staff (psychiatrists and other medical doctors, nurses, nursing auxiliaries, psychologists, physiotherapists, social, educational and administrative staff) were considered. The total number of FTEs and the total number of FTEs allocated to full-time hospitalization were extracted from the SAE database. On a given territory, where both inpatient and outpatient mental health care is coordinated by a single hospital, the FTEs employed in psychiatry and reported in the database are either allocated to full-time hospitalization or to AFTH. FTEs which are not employed in mental health services providing full-time hospitalization are *de facto* employed in services providing AFTH and even FTEs of administrative staff are reported based on the type of care provided by the service they belong to. In addition, considering the overall proportion of FTEs allocated to AFTH allows comparability of data across sectors by adjusting on their overall capacity.

### 2.4. LOS in Full-Time Hospitalization

Our variable of interest, the LOS for each full-time hospitalization, was computed in number of days until discharge using the RIM-P 2012. This was done after obtaining the authorization to access this database from the French data protection authority (CNIL) in July 2013 (Decision DE-2013-077). No informed consent was required from patients as data from the RIM-P is entirely anonymized.

### 2.5. Factors Potentially Associated with the LOS in Full-Time Hospitalization

In addition to the development of AFTH, three types of factors, potentially associated with LOS, were considered as adjustment factors: patients’ characteristics, health care providers’ characteristics and environmental factors.

The patients’ demographic (age and sex) and clinical characteristics (diagnosis) were extracted from the RIM-P database. In accordance with previous research [55], ICD-10 codes were grouped together into broader diagnostic groups (see Table 1). As it is difficult to establish a diagnosis during a single care contact in psychiatry and as comorbidities are frequent [56,57], we considered all diagnoses present in the database for a given patient over the course of the year. To overcome the lack of data on the patients’ socio-economic characteristics in the RIM-P, we created a proxy deprivation index based on the patients’ residential zip codes. We used a validated composite index specifically developed for the French context, called the FDep. This index takes into account the median household income, the percentage of high school graduates in the population aged 15 years and older, the percentage of blue-collar workers in the active population and the unemployment rate, and does not include any health indicator that could lead to circularity [58,59].

Characteristics of sectors and their related hospital were extracted from the SAE database and included legal status (public vs. private non-profit), specialization and participation in teaching activities, as well as organizational factors, such as number of full-time inpatient beds as an indicator of the size of the hospital [44,55,60]. Moreover, the mean value by sector of the patients’ characteristics described above (case-mix) can also have an influence on practice. For example, if a sector treats on average older patients than another sector, this might cause variations in practice. We therefore also considered sectors’ case-mix characteristics.

Finally, environmental factors were extracted from administrative databases and census data [61,62,63,64,65] and calculated for the catchment area of each sector. Those catchment areas, defined as the geographic zone where the sector’s patients originate, were built for each sector after excluding zip codes corresponding to fewer than five patients to avoid bias resulting from a few isolated patients coming from long distances on an occasional basis. The catchment areas were constructed using a geographic information system (Geoconcept^®^ software, Bagneux, France) to convert patients’ text-based zip codes found in the RIM-P into spatial data. Environmental factors included characteristics of the overall health status of the population and the level of urbanization as well as variables related to the supply of additional medical and social care outside of the scope of public psychiatry. Such variables were the availability of inpatient and outpatient psychiatric care provided by the private sector (private for-profit hospitals, self-employed community-based psychiatrists or psychologists and general practitioners) and through social care institutes (residential care or services for disabled individuals) in sectors’ catchment area.

### 2.6. Analysis

#### 2.6.1. Descriptive Analysis

The characteristics of the study population were described either by the mean and standard deviation (SD) or by number (%).

Variations in the LOS in full-time hospitalization and in the development of AFTH between psychiatric sectors were studied by calculating the mean, SD, median, interquartile range, and range of the LOS for each sector and of the development of AFTH for each hospital. A coefficient of variation (CV) [66], which measures the dispersion around the national mean, was computed together with the ratio between the 90th and the 10th percentiles of the distribution, which is less sensitive to outlier values [67].

The association between the variations in the LOS and in the development of AFTH was then assessed through the calculation of the Spearman correlation coefficient.

#### 2.6.2. Multivariate Analysis

To study this association while adjusting for other factors potentially associated with the LOS, we conducted a multivariate analysis with the LOS in full-time hospitalization as a dependent variable. We carried out a natural logarithmic transformation to achieve a more normal distribution given the data skewness. To account for the nested structure of the data, we ran a multi-level model. It was possible to divide variability into four levels: stay, patient, psychiatric sector and hospital. The reliability of hierarchical models however depends on the number of groups [68] and the number of observations per group [69,70,71,72,73,74]. As the mean number of stays per patient and the mean number of psychiatric sectors per hospital were low, two levels were considered: stay/patient level (level 1) and sector/hospital level (level 2).

The development of AFTH was introduced in the model as an explanatory variable as well as the patient, psychiatric sector and environmental characteristics associated with the LOS in the bivariate analyses at a significance level of 0.20 or for which there were strong hypotheses on their association with the LOS. When variables were highly correlated, only one of them was kept in the model based on the strength of association with the LOS and clinicians’ advice. Characteristics of patients were attributed to each of their stays and characteristics of hospitals (in particular the level of development of AFTH) were attributed to each of their sectors, according to the approach used by previous research [75].

To confirm the existence of a random effect at the sector level, we first ran a null model without any explanatory variables (model 1). Second, we introduced the patients’ characteristics (model 2). Third, we added the variables calculated at the sector level (characteristics of the sectors and their environment) (model 3). For each model, we calculated the intraclass correlation coefficient (ICC), which is the proportion of variance that is accounted for by the centre level (i.e., psychiatric sectors), and the proportional change in variance (PCV) to determine the proportion of variance explained by each type of explanatory variable [76]. Finally, we interpreted the value of the estimated regression coefficient associated to the level of development of AFTH (β1) after retransforming the coefficient based on the logarithmic transformation of the dependent variable: %∆ LOS = 100*(e^β1 − 1) [77].

We used a statistical significance level of 0.05 and the analyses were performed using SAS software version 9.3 (SAS Institute Inc., Cary, NC, USA).

## 3. Results

### 3.1. Setting

**Figure 1:**
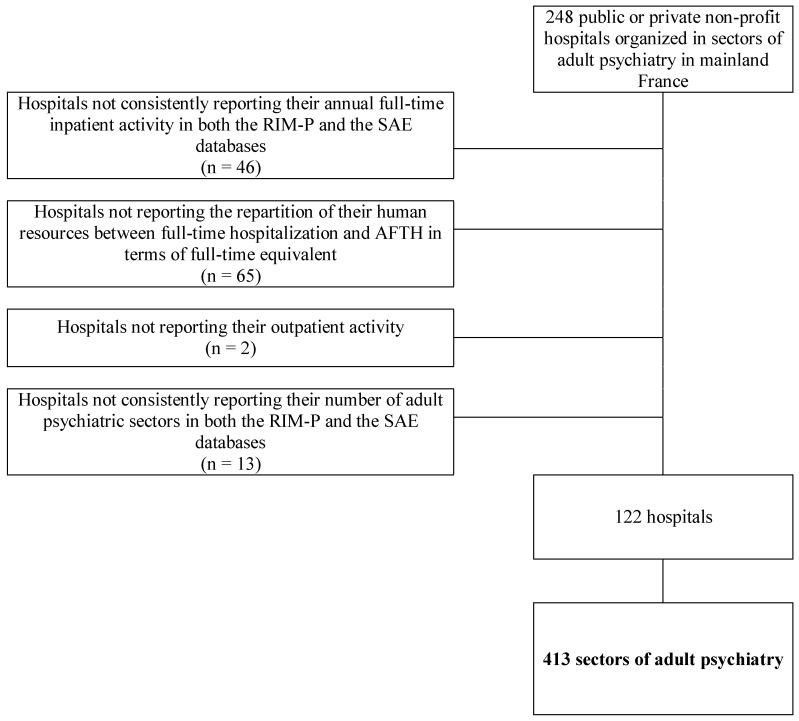
Flowchart for the selection of included sectors based on data quality.

Of the 248 public and private non-profit hospitals participating in public services in mainland France, discharge data from 122 hospitals (49.2%) were included in the analysis based on data quality. These hospitals consisted of 413 sectors of adult psychiatry (see Figure 1) representing 51.4% of all sectors of adult psychiatry in mainland France. Included hospitals did not present any statistically significant differences with excluded ones in terms of main organizational and institutional characteristics or case-mix.

### 3.2. Descriptive Analysis

107,668 patients, matching our diagnostic criteria, were treated in full-time hospitalization in the selected sectors. They accounted for 182,230 stays in full-time hospitalization over the study period and represented 52% of all patients within our diagnosis scope seen in adult psychiatric sectors. The mean age of patients was 46 years (±16) and 54% were female. The two most common diagnoses were mood disorders not including bipolar disorders (27%) and schizophrenia (21%). The mean LOS in full-time hospitalization was 37 days (±72).

Considerable variations between psychiatric sectors were observed both for the LOS in full-time hospitalization and the development of AFTH. The overall mean full-time hospitalization LOS by sector was 36 days and it ranged from 11 to 247.9 days between sectors with a coefficient of variation reaching 60%. These variations were not only a result of sectors with extreme LOS as the ratio between the 90th and the 10th percentiles of the distribution was superior to three (Table 2). The mean value of the ratio of FTEs allocated to AFTH out of the total number of FTEs by hospital amounted to 0.34 (±0.11) and varied between 0.08 and 0.66 among hospitals with a coefficient of variation reaching 33% and a ratio between the 90th and the 10th percentiles of the distribution close to 3 (Table 2).

In the bivariate analysis, a decrease in full-time hospitalization LOS was observed when the level of development of AFTH increased. However, this association was not statistically significant (ϱ = −0.07; *p* = 0.08).

### 3.3. Multivariate Analysis

In the multivariate analysis, we introduced ten individual patient characteristics at level 1. At level 2 we introduced three case-mix characteristics, five institutional or organizational characteristics (in addition to the development of AFTH) of psychiatric sectors, six characteristics of the overall health status of the population in the psychiatric sectors catchment area, as well as eight variables related to the availability of medical and social care in the catchment area (Table 3).

The null model confirmed the existence of a significant centre effect and the necessity to take into account the nested structure of the data (variance = 0.22, *p*-value < 0.0001). Thirteen percent of the total variation in the LOS was related to practice differences between sectors (inter-sector variations) while 87% resulted from differences within sectors linked to case-mix (intra-sector variations) (Table 4).

Patients’ individual characteristics explained 33% of the variations between sectors while sectors’ characteristics explained less than 20% of those variations. The level of development of AFTH was significantly and negatively associated with the LOS (*p* = 0.0493) (Table 5). For each 10% increase of the level of development of AFTH, the LOS in full-time hospitalization decreased by 3.4% (Table 5) when all other patient, health care provider and environmental characteristics were held constant.

Some adjustment factors were also significantly associated with the LOS in full-time hospitalization, in particular characteristics of patients and of the available supply of health and social care outside of public psychiatry. Patient’s age and a female gender were positively associated with the LOS in full-time hospitalization. The presence of a diagnosis of schizophrenia and other psychotic disorders as well as a diagnosis of bipolar or other mood disorders was also associated positively with the LOS. In addition, even if there was not a linear association between patients’ deprivation and LOS, patients in the first and third quintiles of the deprivation index had significantly longer LOS than the most deprived patients (patients in the fifth quintile of the deprivation index). Similarly, some characteristics of patients’ case-mix at the sector level (mean age and percentage of patients suffering from anxiety disorders) were associated with LOS. A positive association was also found for the number of inpatient beds of private psychiatry in the catchment area while the capacity of housing and social rehabilitation centres in the catchment area was negatively associated with the LOS. Among sectors institutional and organizational characteristics, only the level of development of AFTH was significantly associated with the LOS. Finally, the inpatient psychiatric admission rate in the catchment area was negatively associated with LOS while an overall poor somatic health status of the population, approximated by the acute admission rate for somatic disorders for the population in a sector’s catchment area, was positively associated with the LOS (Table 5).

## 4. Discussion

Considerable variations were observed in full-time hospitalization LOS between psychiatric sectors in France. While the majority of those variations resulted from different patients’ characteristics, our results show a significant negative association between the LOS in full-time hospitalization and the development of AFTH after adjusting for a broad range of factors. Our findings are consistent with studies carried out in other local contexts which found that the development of AFTH was associated with a reduced use of inpatient services [28,32]. The considerable variations in the LOS between psychiatric sectors observed in France are also of the same order of magnitude as those underscored by a study carried out on depressed patients in 107 medical centres in the US, which showed that there was a fourfold difference in the mean LOS between the medical centres with the shortest and the longest LOS [78].

Our results provide the first evidence of the benefits of developing AFTH in the French setting as the reduction of the LOS in full-time hospitalization is in alignment with international recommendations for mental health care [13,22,23,24]. They suggest that the development of AFTH can significantly benefit the quality of mental health care in France, taking into account the strong influence of patient and environmental characteristics on LOS. Variables related to the availability of private medical care on sectors’ catchment area (general practitioners community-based private psychiatrists and psychologists or private psychiatric hospitals), which complement the supply of public care provided by psychiatric sectors, were not associated with the LOS in our multivariate analysis. Our main hypothesis is that patients suffering from mental disorders which are seen in private care are not the same patients as those treated in sectorized public psychiatry. They might for instance be wealthier populations (as out-of-pocket costs are higher for private care). This could explain why the availability of private medical care would not influence the LOS of patients seen in psychiatric sectors while the development of AFTH within psychiatric sectors would.

One of the main strengths of our study is the cross-referencing of different data sources, which allowed us to adjust our analysis on a broad range of patient, psychiatric sector and environmental characteristics, using multi-level modelling that has been employed increasingly for the study of factors associated with the LOS in psychiatry [42,55,79,80]. In addition, we did not focus on a limited geographic area but carried out an analysis at the national level which facilitates the generalizability of our results. Additional studies using multiple consensual indicators of quality of care and/or a longitudinal design can now be carried out. Those first findings would in particular be usefully complemented by research aimed at disentangling the mechanisms underlying the impact of AFTH on LOS in the French context. In addition, further work focusing on other variables such as readmission and involuntary admission rates whose reductions are also supported by international recommendations for mental health care [13] will be developed.

The results of our study should however be interpreted in consideration of several limitations linked to the retrospective use of administrative databases, which are considered less accurate than prospective studies, but are the most cost-effective way to gather data on a national scale and allow non-invasive data collection for patients [81]. The RIM-P database was first implemented in 2006 and its limitations have been mentioned previously [21], but it is estimated that since 2010, data quality is sufficient for research purposes [82]. Moreover, whenever possible, we compared data from the RIM-P with data from other databases, such as the SAE to ensure data quality.

However, there are limitations linked to data comprehensiveness as some psychiatric sectors belonging to hospitals where FTEs data were not available could not be included in the analysis, which might limit the external validity of our results. Furthermore, there are some limitations linked to data precision: the share of FTEs allocated to AFTH was calculated on all types of staff while they might not be completely equivalent. In addition, no information was available in the SAE database regarding the distribution of FTEs between the different types of alternatives to full-time hospitalization, while some studies have shown that the impact on the use of inpatient services could vary with the type of AFTH considered [32]. Similarly, there was no information available on the distribution of FTEs between the different forms of care at the psychiatric sector level, while there could be differences between the sectors belonging to the same hospital even if they follow the same general policy.

We were also limited by data availability for the inclusion of adjustment factors potentially associated with the LOS, which could account for some of the unexplained variations in LOS remaining in our multivariate analysis. First, regarding patients’ characteristics, there was no information in the RIM-P database on either symptom or illness severity nor on the socio-economic situation of individual patients. To address this latest limit, we used a deprivation index at the level of patient zip code of residence, following approaches adopted by previous research [83,84,85,86,87,88,89]. Such proxies present the advantage of being mobilizable and operational quickly at a large scale (the national scale in our case) and their use was recently recommended by the French High Council for Public Health whenever individual data are lacking in available information systems [90]. Those limitations result from the use of administrative databases, which however allow the conduction of analyses on a large number of patients (more than 100,000 in our study).

Second, regarding organizational sectors’ characteristics, data was not available on implementation of discharge planning in psychiatric sectors.

Finally, regarding environmental factors related to the availability of medical and social care on the catchment areas of psychiatric sectors, we used data directly available in administrative databases which only provide information relating to a limited number of structures. Detailed mapping of all relevant structures at the local level, similarly to what has been developed in other countries [91,92], should be added to future research to describe with more precision services availability as there are strong hypotheses on its association with the LOS [38,42].

## 5. Conclusions

This study provides the first evidence of the benefits of the development of AFTH at the national level taking into account the particularities of the French system and will provide a lever for policy makers to further its development. One of its main strengths is the cross-referencing of different data sources which allowed us to adjust our analysis on a broad set of patient, psychiatric sector and environmental characteristics. These first results should be supplemented with a focus on other endpoints that may also be impacted by the development of alternatives to full-time hospitalization and are frequently associated with the quality of care, such as unplanned readmission and involuntary admission rates, as well as by studies focusing on other aspects, such as treatment adherence or reduction of caregiver burden. In addition, qualitative research involving the different actors of mental health could usefully add to those first findings.

## Figures and Tables

**Table 1:** Diagnostic groups included in the study.

Diagnostic Group	ICD-10 Wording	ICD-10 Code(s)
Addictions	Mental and behavioural disorders due to psychoactive substance abuse	F10-F19
Schizophrenia	Schizophrenia	F20
Other psychotic disorders	Schizotypal and delusional disorders	F21-F29
Bipolar disorders	Bipolar affective disorders	F31
Other mood disorders	Mood (affective) disorders (except bipolar affective disorder)	F30, F32, F33, F34, F38, F39
Anxiety disorders	Neurotic, stress-related and somatoform disorders	F40-F48
Other mental or behavioural disorders	Behavioural syndromes associated with physiological disturbances and physical factors	F50-F59
	Disorders of adult personality and behaviour	F60-F69
	Pervasive developmental disorders	F84
	Behavioural and emotional disorders with onset usually occurring in childhood and adolescence	F90-F98
	Unspecified mental disorder	F99

**Table 2:** Variations of the LOS in full-time hospitalization and of the development of AFTH between psychiatric sectors.

	Mean (Standard Deviation)	Median (Interquartile Range)	Range	CV (%)	Ratio 90/10th Percentiles
Mean LOS by sector (*n* = 413), days	36.05 (22.30)	30.91 (18.37)	236.91	61.86	3.31
Development of AFTH by hospital hosting each sector (*n* = 122), ratio of FTEs	0.34 (0.11)	0.35 (0.13)	0.58	32.72	2.50

CV: coefficient of variation; AFTH: alternatives to full-time hospitalization; FTE: full-time equivalent.

**Table 3:** Explanatory variables introduced in the multivariate analysis in addition to the level of development of AFTH.

Patient characteristics	Demographic characteristics	Age
		Sex
	Clinical characteristics	Presence of each diagnostic group
	Socio-economic characteristics	Deprivation index
Psychiatric sector characteristics	Case-mix characteristics of patients seen in full-time hospitalization in the sector	Mean age
		% of patients with anxiety disorders
		% of patients with bipolar disorders
	Institutional characteristics of the hospital hosting each sector	Legal status of the hospital
		Specialization in psychiatry
		Participation to teaching activities
		Participation to emergency care
	Organizational characteristics of the hospital hosting each sector	Nb. of inpatient beds per 1000 inh. *
Environmental characteristics	Overall health status of the population (variables computed per 1000 inhabitants of a sector catchment area)	Acute admission rate for somatic disorders
		Mortality rate
		Suicide rate
		Nb. of individuals suffering from long-duration diseases
		% of individuals suffering from psychiatric long-duration diseases among people suffering from long-duration diseases
		Inpatient psychiatric admission rate
	Availability of medical and social care (variables computed per 1000 inhabitants of a sector catchment area)	Nb. of general practitioners
		Nb. of community-based private psychiatrists
		Nb. of psychologists
		Nb. of non-psychiatric inpatient beds
		Nb. of inpatient beds of private psychiatry
		Capacity of housing institutions for disabled individuals
		Capacity of centres providing care through employment
		Capacity of housing and social rehabilitation centres
	Other	Level of urbanization

* The number of full-time inpatient beds per 1000 inhabitants of the catchment area was highly correlated with the total number of sectors per hospital (ρ = 0.90; *p* < 0.0001) and we therefore only introduced the number of beds in the model.

**Table 4:** Estimation of random effects.

	Model 1 (Null Model with No Explanatory Variables)	Model 2 (Model with Individual Patients’ Characteristics)	Model 3 (Model with Individual Patients’ Characteristics and Sectors Characteristics)
Inter-sectors variance (*p*-value)	0.2176 (<0.0001)	0.1466 (<0.0001)	0.1192 (<0.0001)
Standard error	0.0147	0.0101	0.0084
ICC (%)	13.4106	10.3685	8.5972
Δ variance (%)	-	32.6287	18.6903

ICC: intraclass correlation coefficient.

**Table 5:** Estimation of fixed effects in the final model (model 3).

Variable	Modality for Categorical Variable	Estimated Value of the Coefficient	Standard Error	*p*-Value (in Bold When Significant)
Intercept	-	0.7418	0.4377	0.0907
*Explanatory variables introduced at the patient level (level 1)*				
Age	-	0.0077	0.0002	**<0.0001**
Diagnosis of anxiety disorder	1	−0.2058	0.0088	**<0.0001**
	0	0.0000		
Diagnosis of schizophrenia	1	0.1590	0.0089	**<0.0001**
	0	0.0000		
Diagnosis of other psychotic disorder	1	0.2109	0.0090	**<0.0001**
	0	0.0000		
Diagnosis of other mental or behavioural disorder	1	−0.0650	0.0089	**<0.0001**
	0	0.0000		
Diagnosis of addictive disorder	1	−0.0488	0.0092	**<0.0001**
	0	0.0000		
Diagnosis of bipolar disorder	1	0.1957	0.0109	**<0.0001**
	0	0.0000		
Diagnosis of other mood disorder	1	0.0349	0.0082	**<0.0001**
	0	0.0000		
Deprivation index quintile (from lower to higher deprivation)	1	0.0283	0.0129	**0.0285**
	2	0.0212	0.0113	0.0623
	3	0.0262	0.0114	**0.0220**
	4	0.0182	0.0109	0.0969
	5	0.0000		
Female gender	1	0.0000		
	0	−0.0485	0.0063	**<0.0001**
*Explanatory variables introduced at the sector level (level 2)*				
*Characteristics of patients seen in inpatient care aggregated by sector*				
Mean age		0.0080	0.0024	**0.0009**
% of patients suffering from anxiety disorders		−0.0075	0.0016	**<0.0001**
% of patients suffering from bipolar disorders		0.0001	0.0035	0.9892
*Institutional characteristics of the hospital hosting the psychiatric sector*				
Type of legal status of hospital	Private non-profit	0.0612	0.1032	0.5534
	Public	0.0000		
Specialization in psychiatry of the hospital	1	0.0464	0.0477	0.3318
	0	0.0000		
Participation of the hospital to teaching activities	1	−0.1063	0.0563	0.0594
	0	0.0000		
Participation of the hospital to emergency care	1	−0.0979	0.0619	0.1147
	0	0.0000		
*Organizational characteristics of the hospital hosting the psychiatric sector*				
Nb. of inpatient beds in the hospital per 1000 inh.		0.0005	0.0325	0.9890
**Level of development of AFTH**		**−0.0034**	**0.0017**	**0.0493**
*Characteristics of the environment*				
Overall health status of the population of the catchment area (per 1000 inhabitants)				
Acute admission rate for somatic disorders		0.0017	0.0008	**0.0225**
Mortality rate		0.0853	0.0534	0.1105
Suicide rate		0.0010	0.0169	0.9548
Nb. of individuals suffering from long-duration diseases		−0.0015	0.0010	0.1598
% of individuals suffering from psychiatric long-duration diseases among people suffering from long-duration diseases		−0.0235	0.0162	0.1483
Inpatient psychiatric admission rate		−0.1353	0.0471	**0.0042**
Availability of medical care in the catchment area (per 1000 inhabitants)				
Nb. of general practitioners		−0.0726	0.1331	0.5858
Nb. of community-based private psychiatrists		0.3185	0.3460	0.3577
Nb. of psychologists		0.0314	0.0454	0.4898
Nb. of non-psychiatric inpatient beds		0.0020	0.0054	0.7140
Nb. of inpatient beds of private psychiatry		0.2433	0.1157	**0.0360**
Availability of social care in the catchment area (per 1000 inhabitants)				
Capacity of housing institutions for disabled individuals		0.0618	0.0337	0.0669
Capacity of centres providing care through employment		−0.0142	0.0432	0.7420
Capacity of housing and social rehabilitation centres		−0.1169	0.0553	**0.0349**
Level of urbanization				
Level of urbanization (from lower to higher urbanization)	1	−0.0922	0.0578	0.1110
	2	−0.0137	0.0832	0.8693
	3	0.0879	0.2652	0.7406
	4	−0.1259	0.2629	0.6323
	5	−0.1057	0.0618	0.0878
	6	0.0000		
